# The Sick as a Political Asset

**Published:** 1911-07

**Authors:** 


### The Sick as a Political Asset

THE effort to bring about a separation of the county tuberculosis hospital from the county hospital and poorhouse and place it under the control of a board of managers who would conduct it on broad lines for the best interests of its inmates has failed by a vote of the board of supervisors of Erie county who decided, 36 to 13, against separation.

It was expected there would be opposition to the plan of division, but that the supervisors would so boldly and overwhelmingly declare themselves in favor of making a political asset of the sick was not for a moment considered, although that has been one of the tendencies of this branch of the local legislative bodies. There has been no time within the memory of Buffalonians when the board of supervisors has not been devoted to a business administration largely for the benefit of the board itself. In times past it has been a hotbed of political and personal preferment; at the present time there is apparent some leaning toward betterment for there is a number of members who have no stomach for personal aggrandizement at the expense of the indigent sick or those poverty stricken persons who are of necessity compelled to subsist upon the charity of a poorhouse occupancy. For the latter class we can extend only our pity and our sympathy; but for the sick, the tubercular and the physically disabled we can do more. We can demand—and up to the present it has done us little good in our efforts towards betterment of existing conditions—that they be separated from the political activities of a group of personal patriots who for years have snuggled into the best there is of institutional management and control.

The fact that 36 supervisors have defeated the plan for separation of the institutions does not by any means indicate a definite and lasting defeat of the project which is based upon broad and humanitarian lines. The fact that the proposed board of managers was to serve without pay or emolument of any kind may have aroused the sympathy of those of the supervisors who voted against the project. It is difficult for the average supervisor to understand the motive of any citizen who offers to perform a public duty without pay or opportunity to bring to himself some material benefit. He cannot see with his dollar-scaled eye nor can he understand with his vote-littered mind why any human, being should so completely overlook his natural opportunities as to offer his services for the benefit of a charity which has to do with others than himself. With the common run of politicians charity always begins at home and stays there.

In the campaign prior to the temporary defeat of the plan of separation addresses were made before the board of supervisors favoring the separation by Dr. DeLancey Rochester, Dr. R. R. Ross, Charles Stevens and John Coleman, and no breath of criticism was uttered against the administration of either Keeper Bartholomy of the almshouse or of Dr. John Howland, superintendent of the county hospital. The argument was based wholly and entirely upon the necessity for the separation for the benefit of the inmates of the tubercular hospital and yet, in spite of this, chairman Emery in advising against the plan dragged in a wholly unfounded suspicion that medical politics was behind the move and that it were better "to let well enough alone." Mr. Emery comes from East Aurora and is one of that group generally designated as "country members." And for this narrow-minded, unworthy and unjust declaration Mr. Emery was properly rebuked, and with him the other members from beyond the city lines, by supervisors Gaetz and Ladd.

As to the origin of the opposition and the reasons for it one may only surmise without definitely declaring his beliefs; yet it is certain that there has recently developed a business instinct which will be interesting to further investigate and continuously watch and carefully investigate. It is no difficult matter to skim the surface of supervisorial history and note the gradual process of power-pruning which the board has undergone within recent years, beginning with the abolition of the famous purchasing and auditing committee.

One really dislikes to be shorn of power, even the least of us, and it may be that it is an unquenchable thirst for control and authority which has been the occasion of bringing the large majority of the supervisors of today into the unenviable position of making a political asset of the sick; a position which is heartless, disgusting, indecent and wholly shameless.

